# Circularly polarized luminescence from organic micro-/nano-structures

**DOI:** 10.1038/s41377-021-00516-7

**Published:** 2021-04-12

**Authors:** Yongjing Deng, Mengzhu Wang, Yanling Zhuang, Shujuan Liu, Wei Huang, Qiang Zhao

**Affiliations:** 1 State Key Laboratory of Organic Electronics and Information Displays & Jiangsu Key Laboratory for Biosensors, Institute of Advanced Materials (IAM) & Institute of Flexible Electronics (Future Technology), Nanjing University of Posts & Telecommunications (NUPT), 9 Wenyuan Road, 210023 Nanjing, Jiangsu China; 2 Frontiers Science Center for Flexible Electronics (FSCFE), MIIT Key Laboratory of Flexible Electronics (KLoFE), Northwestern Polytechnical University (NPU), 127 West Youyi Road, 710072 Xi’an, Shaanxi China; 3 College of Electronic and Optical Engineering & College of Microelectronics, Jiangsu Province Engineering Research Center for Fabrication and Application of Special Optical Fiber Materials and Devices, Nanjing University of Posts and Telecommunications (NUPT), 9 Wenyuan Road, 210023 Nanjing, Jiangsu China

**Keywords:** Nanoparticles, Optoelectronic devices and components

## Abstract

Circularly polarized light exhibits promising applications in future displays and photonic technologies. Circularly polarized luminescence (CPL) from chiral luminophores is an ideal approach to directly generating circularly polarized light, in which the energy loss induced by the circularly polarized filters can be reduced. Among various chiral luminophores, organic micro-/nano-structures have attracted increasing attention owing to the high quantum efficiency and luminescence dissymmetry factor. Herein, the recent progress of CPL from organic micro-/nano-structures is summarized. Firstly, the design principles of CPL-active organic micro-/nano-structures are expounded from the construction of micro-/nano-structure and the introduction of chirality. Based on these design principles, several typical organic micro-/nano-structures with CPL activity are introduced in detail, including self-assembly of small molecules, self-assembly of π-conjugated polymers, and self-assembly on micro-/nanoscale architectures. Subsequently, we discuss the external stimuli that can regulate CPL performance, including solvents, pH value, metal ions, mechanical force, and temperature. We also summarize the applications of CPL-active materials in organic light-emitting diodes, optical information processing, and chemical and biological sensing. Finally, the current challenges and prospects in this emerging field are presented. It is expected that this review will provide a guide for the design of excellent CPL-active materials.

## Introduction

**Fig. 1:**
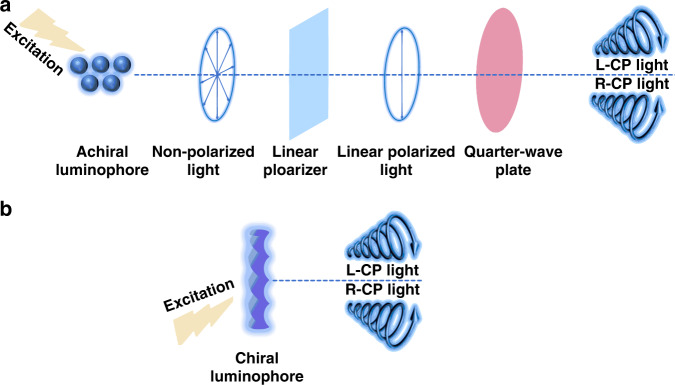
Methods for generating circularly polarized light. **a** Physical method. **b** Circularly polarized luminescence

Polarization refers to the asymmetry between the vibration direction and the propagation direction of the light wave^1,2^. Polarized light can be divided into linearly, circularly, elliptically, and partially polarized light according to different polarization characteristics^3–6^. Among them, circularly polarized light has attracted wide research interests due to its benefits for eye health as well as widespread application prospects in 3D displays, optical sensors, and optical information storage or encryption^7–13^. Traditionally, circularly polarized light can be generated from unpolarized light through the physical method (Fig. 1a). The emitted unpolarized light is first converted into linearly polarized light by the linear polarizer, and then further decomposed into left- or right-circularly polarized light through the quarter-wave plate. During this indirect physical process, at least 50% of energy will be lost^14^. Therefore, it is urgent to develop novel luminescent materials that can directly generate circularly polarized light.

Chirality refers to the geometric property of an object that cannot overlap with its mirror image^15,16^. Chiral molecules usually exhibit unique photophysical properties, such as optical rotatory dispersion, circular dichroism (CD), and circularly polarized luminescence (CPL)^17–20^. CPL means the different emission of left-circularly and right-circularly polarized light in chiral luminophores, and it provides a promising solution to directly generate circularly polarized light with improved efficiency and simplified device structures (Fig. 1b).

CPL-active materials depend on the combination of chiral environments (chiral molecules or asymmetrical environments) and luminescent units. For pure inorganic materials, the chirality of them may be confusing owing to the multiple symmetric and asymmetric relations in the geometry of their atoms^21^. They usually rely on expensive lithography techniques to obtain chirality, and suffer from complex enantiomer separation^22–24^. Organic materials have emerged as promising candidates owing to their easy processing, tunable chiral centers, and excellent photophysical properties^25–27^. Over the past few years, a lot of organic materials have been reported to exhibit CPL activity by combining the chiral units and the luminescent molecules via covalent bonds, such as transition metal complex^28,29^, small organic molecules^30,31^, and conjugated polymers^32,33^. However, they usually suffer from the relatively low luminescence dissymmetry factor (*g*_lum_), which quantifies the asymmetry degree of emission in left- and right-circularly polarized light. The *g*_lum_ is calculated as

1 $$g_{{\mathrm {lum}}} = 2\left( {I_{\mathrm {L}} - I_{\mathrm {R}}} \right)/\left( {I_{\mathrm {L}} + I_{\mathrm {R}}} \right)$$

where *I*_L_ and *I*_R_ mean the luminescence intensity of left- and right-CPL, respectively. The highest |*g*_lum_| of 2 represents completely left- or right-CPL, while *g*_lum_ = 0 corresponds to unpolarized luminescence. Theoretically, the *g*_lum_ can be calculated as

2 $$g_{\mathrm {{lum}}} = 4 \times {\Re} \frac{{\vec \mu \cdot \vec m}}{{|\vec \mu |^2 + |\vec m|^2}}$$

where $\vec \mu$ and $\vec m$ represent the electric and magnetic dipole transition moment, respectively. For organic emitters, the transitions are usually electric dipole allowed and magnetic dipole forbidden. The negligible $|\vec m|$ and high $|\vec \mu |$ result in poor |*g*_lum_| (10^−5^–10^−3^), which limits the practical applications^34,35^. For the application of CPL materials, high |*g*_lum_| is the prerequisite. The higher |*g*_lum_| means the better polarization degree of the emitted light, which refers to the lower energy loss. Thus, exploring new approaches to constructing CPL-active materials with high |*g*_lum_| is a vital issue.

The development of nanotechnology and nanophotonics provides inspiration and feasibility for harvesting high |*g*_lum_|. Compared with the single organic molecule, organic micro-/nano-structures usually exhibit integrated or even amplified functions^36,37^. In terms of CPL, the |*g*_lum_| of organic micro-/nano-structures can generally be amplified with one or two orders of magnitude due to the highly ordered molecular stacking, and some theoretical investigations reveal that the CPL dissymmetry might depend on the modulation sum over the exciton coupling on the aggregation structures^26,35,38,39^. In addition, the aggregation structures provide a platform to combine multiple components for the purpose of producing the integrated or synergetic functions via convenient intermolecular non-covalent interactions rather than complicated covalent synthesis. For example, CPL activity can be realized by co-assembly of achiral luminophores with non-emissive chiral molecules^40,41^. Moreover, the optical properties of micro-/nano-structures depend not only on the properties of the building blocks themselves but also on their arrangement. Achiral luminophores can obtain CPL activity by spontaneous symmetry breaking during the assembly process^42,43^.

At present, the research on CPL-active organic micro-/nano-structures is just in its infancy but rapidly developing, and the potential applications are emerging in many areas. This review focuses on the latest progress of organic micro-/nano-structures with CPL activity, aiming to provide a comprehensive insight into the relationship among molecular designs, assembly structures, and chiroptical properties. Firstly, we highlight the design principles of various micro-/nano-structures and the approaches for regulating CPL signals. Then, we introduce the applications of CPL-active materials in organic light-emitting diodes (OLEDs), optical information processing, and chemical and biological sensing. Finally, we will present the challenges and perspectives in this emerging field.

## CPL-active organic materials with micro-/nano-structures

Unlike inorganic materials, organic materials are soft and flexible. Therefore, the hard top-down strategy is difficult to be applied to construct organic micro-/nano-structures. Fortunately, self-assembly, as a bottom-up method, provides a facile and universal method for constructing micro-/nano-structures through intermolecular non-covalent interactions. So far, various CPL-active organic micro-/nano-assemblies have been developed, and the highest |*g*_lum_| of the assembly has exceeded 1.4, which is close to the theoretical maximum^44^. In this section, we will introduce several typical examples: (1) self-assembly of small molecules; (2) self-assembly of π-conjugated polymers; and (3) self-assembly on micro-/nanoscale architectures (Table S1).

### Self-assembly of small molecules

For micro-/nano-structures assembled from organic small molecules, there are three approaches to realizing CPL activity: (1) self-assembly of chiral luminescent molecules; (2) co-assembly of achiral luminescent molecules with chiral molecules; and (3) symmetry breaking of achiral luminescent molecules.

**Fig. 2:**
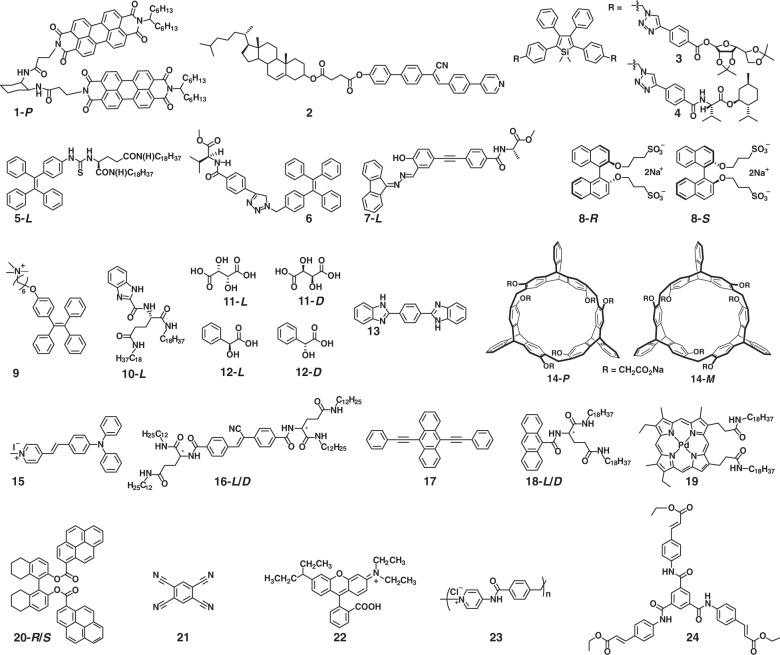
Chemical structures of small molecule assembly system

Compared with the single molecular state, the assembly structures of chiral luminescent molecules usually possess the relatively amplified |*g*_lum_| owing to the enhanced exciton couplings. For example, chiral perylene bisimide derivative **1** (see Fig. 2) self-assembled into helical fibers in methylcyclohexane through π–π interaction^38^. The assemblies exhibited red-shifted absorption, weakened emission, and longer emission lifetime at long-wavelength region than that of the monomer dispersed in chloroform, which indicated the enhancement of exciton coupling. Excitingly, the assemblies exhibited significantly increased |*g*_lum_| (from 8 × 10^−3^ to 2.5 × 10^−2^) benefiting from the effective long-range exciton coupling. Although self-assembly can amplify |*g*_lum_|, it usually leads to significant fluorescence quenching, because the excited state energy was dissipated by the non-radiative transition pathways. Fortunately, aggregation-induced emission (AIE) provides an approach for achieving both high |*g*_lum_| and high efficiency^45,46^. For example, a chiral AIE molecule **2** was synthesized by combining cyanostilbene and chiral cholesterol group^47^. No CD signal was detected in the monomer state due to the limited chirality transfer between the chiral centers and chromophores. Through the heating–cooling process in DMSO solution, compound **2** could self-assemble into the gel state with nanohelix structures through strong intermolecular π–π interactions, resulting in chirality transfer from the chiral center to the assemblies. Thus, both CD and CPL signals were observed in the gel state with enhanced emission. Similarly, various AIE systems (**3**–**7**) have been utilized to trigger aggregation-induced CPL phenomena, such as silole derivatives^48,49^, tetraphenylethylene derivatives^50,51^, and Schiff base groups^52^.

Although self-assembly of chiral luminescent molecules is an effective and universal approach to amplifying |*g*_lum_|, it still requires complicated covalent synthesis to combine the chiral units and the luminescent molecules. The co-assembly of achiral luminescent molecules with chiral molecules provides a convenient approach via intermolecular non-covalent interactions. For example, by co-assembly of tetraphenylethylene derivative **8** and chiral binaphthyl derivatives **9**, the nanofibers could be formed through the electrostatic interactions, and the solid-state helical fibers showed excellent CPL performance^53^. The chirality of the assemblies came from the chirality transfer between the chiral center and the assemblies, and the electrostatic interactions were found to facilitate the chirality transfer. Furthermore, various non-covalent interactions can also be employed to promote the chirality transfer (**10**–**15**)^54–56^, such as coordination bonds, hydrogen bonds, π–π interaction, and host–guest interaction.

**Fig. 3:**
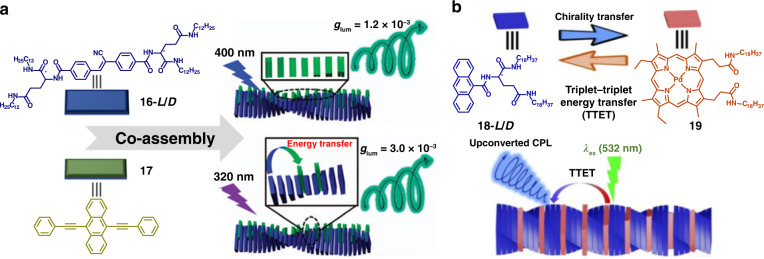
Schematic diagram of energy transfer for amplifying CPL. **a** CPL from Föster resonance energy transfer in the co-assembly systems. **b** Uconverted CPL through triplet-triplet energy trsnsfer. Images reprinted with permission from: **a** ref. ^57^ from Springer Nature; **b** ref. ^60^ from Wiley

In the co-assembly systems, the energy and charge transfer are found to be beneficial for amplifying the |*g*_lum_|. As shown in Fig. 3a, chiral donor π-gelator **16** and achiral π-acceptor **17** co-assembled into nanohelix structures^57^. The |*g*_lum_| of assemblies by exciting the donor at 320 nm was larger than the obtained by directly exciting the acceptor at 400 nm. This interesting phenomenon was attributed to the efficient Föster resonance energy transfer under excitation at 320 nm. Triplet–triplet annihilation based photon upconversion is another promising energy transfer approach, in which the non-radiative transition might decrease the $\left| {\vec \mu } \right|$. Meanwhile, electron spin polarization might take place, which could amplify the $\left| {\vec m} \right|$. The synergy of amplified $\left| {\vec m} \right|$ and depressed $\left| {\vec \mu } \right|$ resulted in the increase of |*g*_lum_|^58,59^. In the co-assembly system of chiral anthracene derivative **18** and achiral sensitizer **19** (Fig. 3b), triplet–triplet energy transfer can occur simultaneously from the donor **19** to the acceptor **18** ^60^. By exciting the donor at 532 nm, the co-assemblies exhibited upconverted CPL at 460 nm. Moreover, charge transfer systems have been found to possess forbidden electron dipole transition and relatively large magnetic dipole transition. Chiral emissive molecule **20** and achiral electron acceptors **21** with matched energy levels were selected to construct the co-assembly system^61^. The |*g*_lum_| of the co-assemblies was almost 200 times that of chiral donor alone, which benefited from the large $\left| {\vec m} \right|$ and small $\left| {\vec \mu } \right|$ in the charge transfer states.

Apart from the two approaches mentioned above, in which the chiral units are necessary, self-assembly provides a possibility to endow exclusively achiral luminescent molecules with CPL activity by spontaneous symmetry breaking. The first report about CPL from exclusively achiral molecule was the co-assembly of rhodamine B (**22**) and achiral ionic polymer **23**^62^. Unfortunately, the CPL could only be detected during the stirring process. Subsequently, an achiral C_3_-symmetric molecule **24** was designed to exhibit CPL behavior without any chiral additives or asymmetric environments^42^. Although compound **24** was achiral, it could form supramolecular gels with unequal amount of left- and right-handed nanotwists. Interestingly, strong CPL was observed in the gels with |*g*_lum_| up to 8 × 10^−3^.

In general, self-assembly of organic small molecules provides exciting approaches to constructing CPL-active organic micro-/nano-structures, and the |*g*_lum_| can be further amplified by various strategies, particularly the energy and charge transfer.

### Self-assembly of conjugated polymers

CPL-active conjugated polymer assemblies have drawn great attention owing to their well-defined structure modification, tunable luminescent properties, and easy processing^63,64^. They can be constructed through the following two approaches: (1) self-assembly of chiral conjugated polymers, including main-chain chiral conjugated polymers and conjugated polymers with chiral side-chain; (2) doping achiral conjugated polymers with chiral additives.

**Fig. 4:**
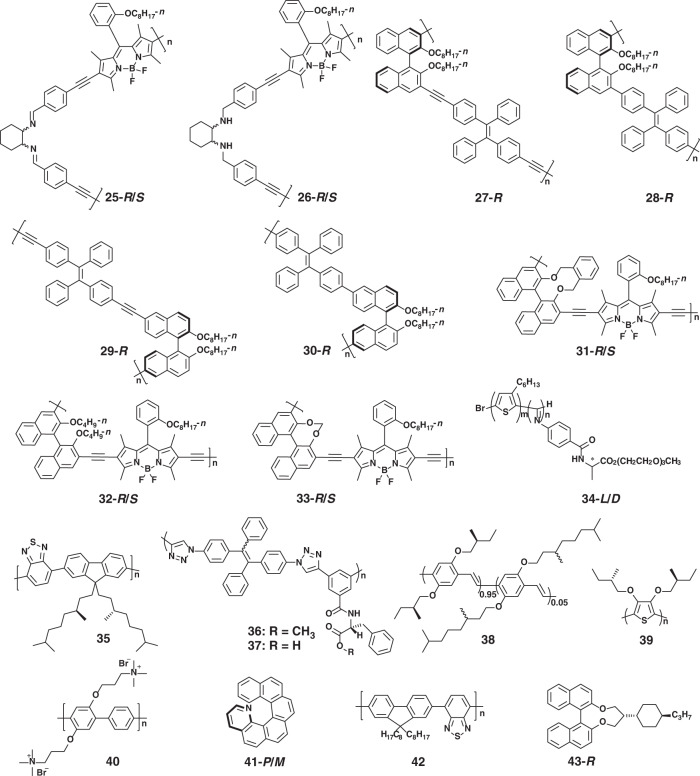
Chemical structures of polymer-assembly systems

Main-chain chiral polymers can be obtained by introducing chiral copolymer units into the polymer backbones. For example, two pairs of chiral polymer enantiomers (**25** and **26**, see Fig. 4) were synthesized by employing cyclohexanediamine derivatives as chiral units^65^. All of them exhibited similar absorption and fluorescence behavior, but only polymer **25** showed obvious mirror-image CD and CPL signals. This might be the result of the chirality transfer from cyclohexanediamine to chromophores through the extended conjugated main-chain backbone. In contrast, no CD or CPL could be detected for polymer **26** due to the lack of conjugated linkers between the chiral center and aromatic group. This work indicates that the conjugation degree of the main chain plays an important role in CPL performance. In order to maintain the π-conjugated structures, axial chiral molecules represented by 1,1′-binaphthol have been used as ideal copolymerization units. For instance, a series of main-chain chiral conjugated polymers (**27**–**30**) were designed and synthesized by co-polymerization of (*R*)-1,1′-binaphthyl and tetraphenylethene units^66^. All these four polymers showed CD signals in the region under 300 nm, which were closed to monomeric model compounds. Among them, polymer **27** displayed CD signals in the region of 350–430 nm. This might be because polymer **27** performed the helical configuration in the conjugated polymer chain. Not only that, polymer **27** exhibited obvious CPL in aggregation state due to the formation of helical nanofibers and the typical AIE phenomenon, while no CD or CPL could be detected for the other three polymers. These results indicate that conjugated linkers and the linked positions significantly affect the microstructures and chiroptical properties of aggregates. In addition, the dihedral angles of binaphthyl moieties also affect the CPL signals. In chiral-conjugated polymers (**31**–**33**), they exhibited different dihedral angles owing to the different steric hindrance^67^. Interestingly, the |*g*_lum_| gradually increased as the decrease of dihedral angle.

However, the development of main-chain chiral-conjugated polymers has been restricted due to the limited axial chiral units and the unpredictable CPL properties. Decorating the conjugated polymer with chiral pendants provides an alternative choice, and various CPL-active conjugated polymer systems (**34–39**) have been developed^64,68–70^, where the chiral pendants play an important role in the arrangement of the polymer chains. Typically, chiral π-conjugated block copolymers **34** composed of poly(3-hexylthiophene-2,5-diyl) (P3HT) and poly(phenyl isocyanide) (PPI) with chiral substituent were prepared^64^. Employing the crystallinity of P3HT and the chirality of PPI, the approach of crystallization-driven asymmetric self-assembly was developed to construct helical structures. Interestingly, the chirality of side-chain could be transferred to the supramolecular assemblies during the asymmetric self-assembly process, resulting in the enhanced CPL, and the |*g*_lum_| of assemblies could be regulated by tuning the helicity and length of the helical nanofibers.

The most promising approach to constructing CPL-active conjugated polymer assemblies is doping achiral π-conjugated polymer with chiral additives, such as biaryl compounds and helicene. The CPL-active spherulites were reported by doping axially chiral binaphthyl derivative **8** into π-conjugated polyelectrolyte **40**^71^. The interchain helically π-stacked structure was formed through the π–π interactions, and further hierarchically assembled into spherulites stabilized by the electrostatic interactions. Owing to the chirality transfer from chiral additive to achiral π-conjugated polymer, the spherulites exhibited blue CPL with a |*g*_lum_| of ~10^−2^. Chiral additives with high helical twisting power can induce polymers to form helical conformation. Helicene enantiomer **41** was doped into the achiral polymer **42** with different ratios^72^. As expected, there was no CD response for the pure polymer. However, both CD and CPL responses were enhanced with the increase of the doping content, and the maximum value of |*g*_lum_| reached 0.5. Subsequently, by doping a non-emissive chiral dopant with high helical twisting power **43**, the |*g*_lum_| up to 0.72 was realized by the twisted stacking of achiral-conjugated polymer **42**^73^.

### Self-assembly on micro-/nanoscale architectures

Pre-existing micro-/nano-structures can serve as templates to obtain CPL-active micro-/nano-assemblies with specific functions by assembling active molecules into the pores or surface of the templates. Such a method has been considered to be a simple and effective strategy for preparing micro-/nano-structures due to its high preparation efficiency, regular morphologies, and good structural stability. According to the properties of the templates, it can be divided into achiral nanotemplates and chiral nanotemplates.

#### Achiral nanotemplates

**Fig. 5:**
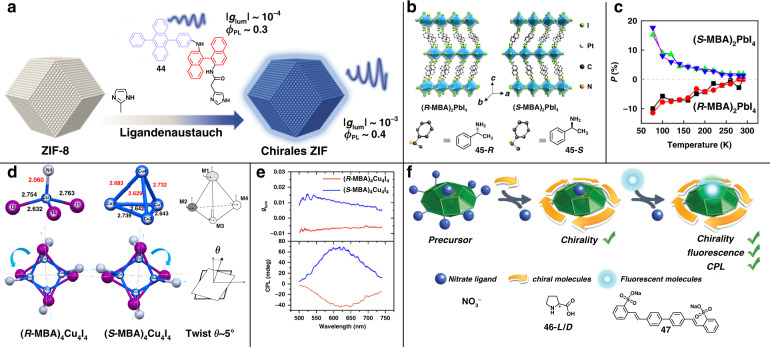
CPL from the assembly of chiral ligands on achiral nanotemplates. **a** Amplifying CPL by assembly the chiral luminescent molecule onto the achiral ZIF-8 skeleton. **b** Schematic illustrations of crystal structures of (*R*/*S*-MBA)_2_PbI_4_. **c** The influence of temperature on the photoluminescence polarization of (*R*/*S*-MBA)_2_PbI_4_. **d** The configuration features of (*R*/*S*-MBA)_4_Cu_4_I_4_. **e** The CPL spectra of (*R*/*S*-MBA)_4_Cu_4_I_4_. **f** Schematic diagrams of the stepwise surface modification for Ag(I) clusters. Images reprinted with permission from: **a** ref. ^77^ from Wiley; **b**, **c** ref. ^82^, **d**, **e** ref. ^92^ from ACS; **f** ref. ^93^ from Wiley

Metal-organic frameworks (MOFs) consisting of metal nodes and bridged organic ligands have received considerable attention due to their regular morphologies and unique porous structures^74^. MOFs can serve as ideal nanotemplates to deposit or encapsulate luminescent molecules into the surfaces or pores, resulting in improved luminous performance^75–78^. As shown in Fig. 5a, the enhanced CPL has been realized by reorganizing chiral binaphthyl-derived emitters **44** onto the achiral zeolitic imidazolate framework (ZIF-8)^77^. Although the chirality inversion occurred owing to the change of dihedral angle in the axially chiral ligands, the |*g*_lum_| of chiral emitters exhibited significant amplification (from 10^−4^ to 10^−3^) due to the well-ordered arrangement of chiral emitters on the skeleton of ZIF-8. Furthermore, the assemblies showed enhanced emission efficiency because the rigid structures of ZIF-8 can restrict the intramolecular motions to suppress non-radiative transition. This work provided a feasible approach for achieving both high |*g*_lum_| and high efficiency in CPL materials.

Hybrid organic–inorganic perovskites (HOIPs) have become a research hotspot in optoelectronic field, due to their direct band-gaps and high quantum efficiencies^79^. HOIPs have the general formula of ABX_3_, where A is monovalent organic cation, B is divalent metal cation, and X is halogen anion^80^. Benefiting from the excellent luminescence property, tunable hybrid compositions, and flexible crystal structures, HOIPs provide a versatile platform to realize CPL activity by introducing chiral ligands as the A-site cation^81–83^. For example, a chiral two-dimensional (2D) perovskite was synthesized by incorporating chiral methylbenzylamine **45** (*R*/*S*-MBA) as the A-site cation (Fig. 5b), and the maximum |*g*_lum_| of 0.352 was detected at 77 K ^82^. Unfortunately, the degree of polarization gradually decreased with increasing temperature (Fig. 5c). This might be attributed to the reduced lattice distortion arisen by enhanced electron–phonon couplings and thermal-expansion interactions.

Ligand-protected metal clusters, consisting of metal core surrounded by organometallic motifs, are a new type of luminescent nanomaterial, which have attracted wide research interests due to its unique quantum size effect^84,85^. The diameter of clusters is usually <2 nm, close to the Fermi wavelength of electrons. The quasi-continuous energy levels break up into discrete electronic states, allowing the electronic transition, resulting in large Stokes shift, high quantum efficiency, and good photostability. Inspired by the excellent luminescence property and diversity of organic ligands, nano-sized metal clusters, especially Cu clusters, Ag clusters, and Au clusters, provide excellent opportunities to combine chirality and luminescence by introducing chiral ligands, such as chiral amine, chiral phosphine, chiral thiolate, and chiral alkynyl^86–91^. For example, a pair of enantiomeric Cu clusters was successfully synthesized by introducing *R*/*S*-MBA^92^. The Cu cluster skeleton exhibited distorted cubane-like motifs with the helical twist angle about 5° (Fig. 5d), which was an important factor that determines the chirality of the Cu clusters. These clusters showed intense orange emission with high quantum efficiency (~60%), and the *g*_lum_ were about 1.0 × 10^−2^ and −6 × 10^−3^, respectively (Fig. 5e).

Using a stepwise surface modification approach, the CPL activity has been successfully achieved on Ag(I) clusters^93^. The anionic nitrate ligand was gradually replaced by the chiral amino acid **46** and achiral luminescent ligand **47**, while the original Ag(I) cage structure was nearly maintained (Fig. 5f). Benefiting from the combination of chirality and luminescence, the chiral Ag(I) clusters exhibited CPL response with |*g*_lum_| about 5 × 10^−3^. This post-modification method opens a new approach to constructing CPL-active metal clusters.

#### Chiral nanotemplates

Taking advantage of the space-confined and structure-directing effect of pre-existing chiral nanostructures, CPL can be generated from the induced chirality by encapsulating achiral luminophores into the chiral cages of nanostructures, where chiral nanostructures serve as templates^94–96^.

**Fig. 6:**
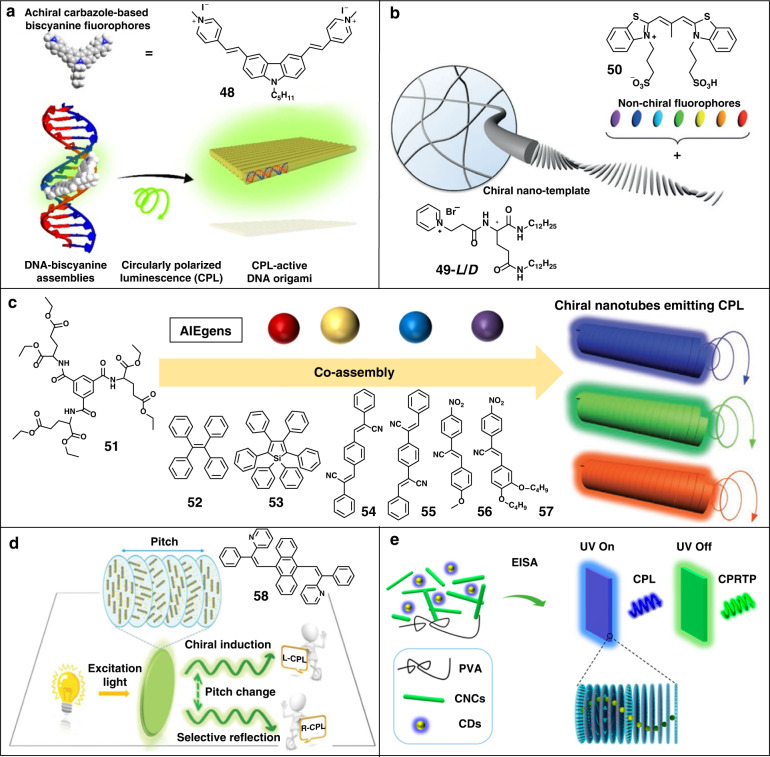
CPL from the induced chirality of chiral nanotemplates. **a** Schematic illustration of DNA–biscyanine hybrid CPL-active materials. **b** Schematic illustration of induced CPL by encapsulating achiral dyes into chiral gelator. **c** Schematic illustration of induced CPL by encapsulating AIE dyes into chiral gelator. **d** Schematic illustrations of the selective reflection and chiral induction in CNCs. **e** Schematic illustrations of the generated CP-RTP. Image reprinted with permission from: **a** ref. ^97^ from ACS; **b** ref. ^98^, **c** ref. ^99^ from Wiley; **d** ref. ^102^ from RSC; **e** ref. ^103^ from ACS

As shown in Fig. 6a, DNA molecules, natural chiral molecules with unique duplexes structures, were employed as chiral nanotemplates^97^. The achiral cyanine dye **48** was anchored into the chiral DNA molecules. The chiral transfer could occur from the natural chirality of DNA molecules to the bound dye molecule, inducing a remarkable CPL activity from the assemblies. At the same time, the intramolecular rotation of dye molecule was restricted by the electrostatic interaction with DNA templates, resulting in the enhanced emission. This work provides an inspiration to induce CPL activity employing bio-macromolecules.

Chiral gelators which can spontaneously form supramolecular gels with micro-/nano-structures through surface-tension effects provide convenient templates. As shown in Fig. 6b, glutamic acid derivatives **49** were first synthesized to provide chiral ordered nanotemplates^98^. By introducing achiral fluorescent dye **50**, CPL signal was generated due to electrostatic interactions between the dye and the template, as well as the enhanced chirality of the template. Subsequently, the CPL activity of the condensed phase was obtained by co-assembly of a chiral gelator **51** and achiral AIE luminophores (**52**–**57**) (Fig. 6c)^99^. During assembly, the host gelator spontaneously formed nanotubes where AIE luminophores were confined in the nanotubes. Induced by chiral spaces of the gel nanotube, achiral AIE molecules exhibited enhanced emission and excellent CPL performance with |*g*_lum_| of about 10^−3^. Besides, full-color circularly polarized emission could be easily achieved by simply changing the doped dyes.

Cellulose nanocrystals (CNCs) can self-assemble into left-handed chiral nematic structures, which can selectively reflect circularly polarized light. When the wavelength of incident light matches with the photonic band-gaps (PBGs), left-handed circularly polarized light will be selectively reflected, whereas right-handed circularly polarized light will be transmitted. The reflected wavelength can be approximated by

3 $$\lambda = nP\cos \theta$$

where *n* represents the average refractive index, *P* means the helical pitch, and *θ* is the angle between the incident light and the helix axis^100^. In addition, the overlap between the fluorescent emission and the PBGs can lead to right-handed CPL emission. Owing to the selective reflection, dissymmetry factors of −0.87 for passive right-CPL and 0.29 for passive left-CPL were detected when 512 nm of incident light was applied onto neat chiral photonic CNCs films (PBGs peak wavelength at 510 nm). When luminophores whose emission maxima matched with the PBGs were doped into chiral photonic CNCs films, the spontaneous emission of luminophores was transformed into right-CPL emission with |*g*_lum_| up to 0.68 ^101^.

Chiral induction is another feature of CNCs. As shown in Fig. 6d, the induced CPL could be generated via the co-assembly of achiral luminophore **52** and CNCs^102^. When the helical pitch of the film was about 530 nm, the composite film exhibited right-CPL owing to the selective reflection and PBGs effect. Surprisingly, the composite film with a helical pitch of 660 nm showed left-CPL with a *g*_lum_ of 3.3 × 10^−2^. That might be attributed to the induced chirality and suppressed PBGs effects because the PBGs were far away from the fluorescence emission wavelength. Recently, circularly polarized room-temperature phosphorescence (CP-RTP) was realized by co-assembly of CNCs, polyvinyl alcohol (PVA), and carbon dots (Fig. 6e)^103^. The PBGs could be regulated by the ratio of CNCs/PVA, resulting in tunable CPL with invertible handedness. At the same time, multiple hydrogen bonds of the hybrid chiral photonic films prevented the non-radiative relaxation and stabilized the triplet excitons produced by the intersystem crossing of carbon dots, resulting in CP-RTP. The dissymmetry factor (*g*_RTP_) changed from −0.461 to 0.036 with the red-shifted PBGs, and the lifetimes up to 103 ms.

In summary, self-assembly on micro-/nanoscale architectures provides a convenient and efficient approach for constructing CPL-active materials due to the well-defined morphology, structure and arrangement. Especially, induced chirality from chiral micro-/nano-structures would be the most promising approach to producing a diversity of CPL materials.

## Regulation of CPL through external stimuli

The formation of assemblies is driven by intermolecular non-covalent interactions. This process is dynamic and sensitive to external stimuli, such as solvent, pH value, metal ions, mechanical force, and temperature. This provides an opportunity to regulate the chirality and CPL signal in self-assembled systems.

### Solvent

**Fig. 7:**
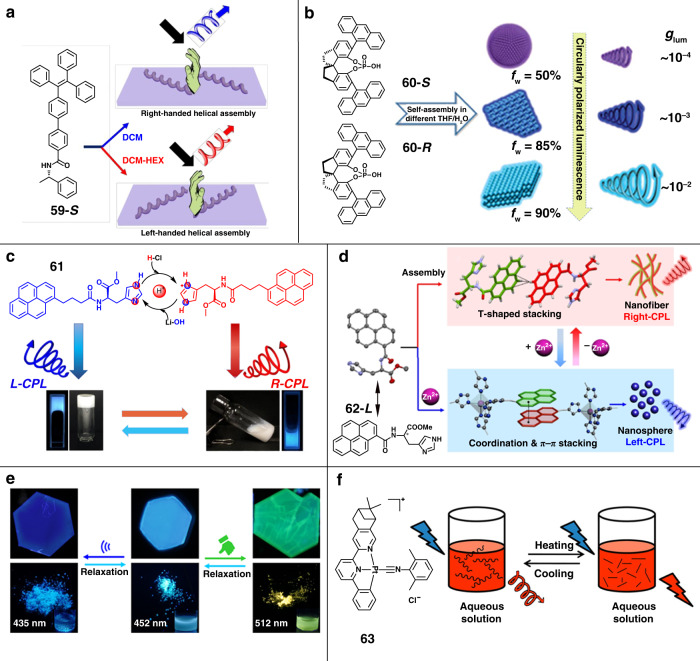
Schematic illustrations of CPL regulation through different external stimuli. **a** Solvent polarity. **b** Solubility. **c** pH value. **d** Metal ions. **e** Mechanical force. **f** Temperature. Images reprinted with permission from: **a** ref. ^104^, **b** ref. ^35^ from RSC; **c** ref. ^105^ from ACS; **d** ref. ^107^, **e** ref. ^110^ from Wiley, **f** ref. ^111^ from ACS

Solvent, the medium of the self-assembly process, can significantly influence the self-assembly process through specific solute–solvent interactions. Therefore, adjusting the solvent properties can control the supramolecular chirality of assembly structures, such as polarity, concentration, and so on. Helicity inversion has been realized by changing the solvent polarity without changing the molecular chirality^104^. As shown in Fig. 7a, chiral tetraphenylethylene derivative **59** was synthesized by linking the fluorescent group with chiral phenethylamine, where amide linkage provided flexible conformational rotation. In dichloromethane solution, a positive CPL signal was observed, and CPL intensity decreased with the increase of hexane volume ratio. Once the volume ratio of hexane up to 80%, CPL inversion occurred, and the negative CPL signal was enhanced with the further increase of hexane content. To illustrate this interesting CPL inversion, the possible molecular packing models were proposed. In dichloromethane solution, the molecule performed *cis*-like conformations and formed right-handed helical assemblies. By adding hexane, the solution polarity decreased, altering the C–C bond rotation between two phenyl groups, which plays a significant role in molecular packing. When the volume ratio of hexane up to 80%, the molecule performed *trans*-like conformations and followed head-to-tail arrangement to form left-handed helix.

Morphology-dependent CPL activity has been reported by adjusting the solvent compositions (Fig. 7b)^35^. By adjusting the content of poor solvent, chiral luminescent molecules **60** could self-assemble into different dimensional morphologies. The |*g*_lum_| of the system was found to be gradually amplified with aggregate structures changed from 0D nanospheres to 2D and 3D nanoflakes, and the *g*_lum_ of 3D stacked nanoflakes reached 0.029. Through experiments and Materials Studio simulations, the enlarged *g*_lum_ might be attributed to intense excimer emission induced by strong intermolecular interaction in more ordered structures.

### pH value

The pH value can significantly affect the intermolecular hydrogen bonds, which may cause different arrangements of molecules, triggering the inversion of helicity. Inspired by the proton shuttle of histidine, the reversible conversion of supramolecular chirality and CPL was realized (Fig. 7c)^105^. Pyrene-functionalized l-histidine gelator **61** was synthesized, and it self-assembled into short nanosheet structures with left-CPL through hydrogen bonds and π–π stacking. When adding proton acid, the protonation of imidazole resulted in the disappearance of hydrogen bonds between the amide and imidazole group, and the orientation of pyrene chromophores was reversed, leading to the change of the morphology and CPL inversion. By adding an external base, the protonation process could be deprotonated, leading to the recovery of the morphology and CPL signal. And this reversible protonation–deprotonation process could be repeated at least five cycles, which showed excellent repeatability.

### Metal ions

Coordination interaction was widely applied in supramolecular self-assembly because of its unique directionality and reversibility. The introduction of metal ions can alter multiple non-covalent interactions, which possibly drive self-assembly in different pathways^54,106,107^. Chiral pyrene-conjugated histidine **62** was designed and synthesized, which could self-assemble into nanofibers with right-CPL (Fig. 7d)^107^. Crystal structures indicated that two pyrene rings form the dimer via intermolecular CH–π interaction or T-type stacking, and further self-assembled into nanofibers. Once coordinating of PyHis with Zn^2+^, a different assembly pathway was driven by synergistic effects of coordination and π–π interactions, leading to the nanofiber transformed into nanospheres with the inversion of CPL. When ethylene diamine tetraacetic acid was added as a competing ligand, the assembly morphology and CPL signal could be reversed. Through the dynamic coordination-dissociation strategy, multiple reversible switching of CPL could be realized around four cycles, providing new ideas for the development of smart responsive CPL materials.

### Mechanical force

In the aggregate state, the properties of materials depend not only on the molecular structure but also on the molecular stacking^36^. Typically, mechanochromic materials can change their absorption or emission properties (wavelength, intensity, or lifetime) when the molecular stacking modes are changed under mechanical forces^108^. The CPL signal can also be changed under mechanical forces, owing to the change of molecular stacking. Recently, two chiral Au(I) complexes with mechanochromic behaviors were synthesized^109^. When the mechanical force was applied, the powder exhibited turn-on phosphorescence behavior with the transformation of morphology from isolated crystallites to well-defined microcrystals. Additionally, CPL would arise with |*g*_lum_| of 4 × 10^−3^. Such changes might be attributed to the sliding of molecular stacking driven by the synergistic non-covalent interactions. A dual mechano-switchable CPL was achieved via ultrasonication and grinding (Fig. 7e)^110^. CPL-active MOFs were constructed from achiral tetrakis(4-pyridylphenyl)ethylene and chiral camphoric acid through the chiral reticular self-assembly with Cd^2+^. Ultrasonication or grinding could trigger the local or global dynamics of the AIEgen rotors in the chiral frameworks, which caused the different rotation or stacking mode of AIEgen rotors. Blue and red-shifted CPL was obtained after ultrasonication and grinding, respectively. Notably, mechanical force is usually destructive. Therefore, these changes could only be recovered by relaxing overnight in DMF solution.

### Temperature

The intermolecular interactions are thermodynamic unstable and the strength of non-covalent bonds is temperature-dependent. Therefore, the assemblies are usually sensitive to temperature. As shown in Fig. 7f, pinene functionalized cyclometalated Pt(II) complex **63** showed temperature-dependent CPL signals^111^. In aqueous solutions, the molecules were associated through Pt–Pt, π–π, and hydrophobic–hydrophobic interactions to form the one-dimensional helical chain structures, resulting in the detectable CPL signals. Interestingly, the CPL activities were lost upon increasing the temperature from 295 to 353 K and recovered upon cooling to 295 K owing to the reversible intermolecular interactions.

Various types of external stimuli have been developed to regulate the CPL performance of assemblies, and these regulation processes are usually dynamic, exhibiting great potential in smart CPL-switches. In order to satisfy the requirement of practical applications, further studies about the response time, hysteresis, and repeatability are demanded.

## Applications of CPL-active organic micro-/nano-structures

As mentioned above, CPL-active organic micro-/nano-structures can harvest both high quantum efficiency and high |*g*_lum_|, which make them good candidates for potential applications in 3D displays, optical information processing, and sensing.

### CP-OLEDs

For OLEDs, in order to obtain high image contrast, it is usually necessary to use a circular polarizer to reduce the reflectivity of the surrounding environment. Thus, only half of the emitted light can reach the eyes, causing great loss of brightness and energy efficiency. Circularly polarized OLEDs (CP-OLEDs), showing the direct emission of circularly polarized light with the same handedness as the circular polarizer, can prevent energy loss, resulting in lower power consumption^14,112^. Since the first CP-OLEDs were reported in 1997^113^, various CP-OLEDs have been developed. So far, the highest electroluminescent asymmetry factor (*g*_EL_) was reported in 2017^73^. By doping a non-emissive chiral dopant with high helical twisting power, the achiral conjugated polymer displayed twisted stacking with high |*g*_EL_| of 1.13.

**Fig. 8:**
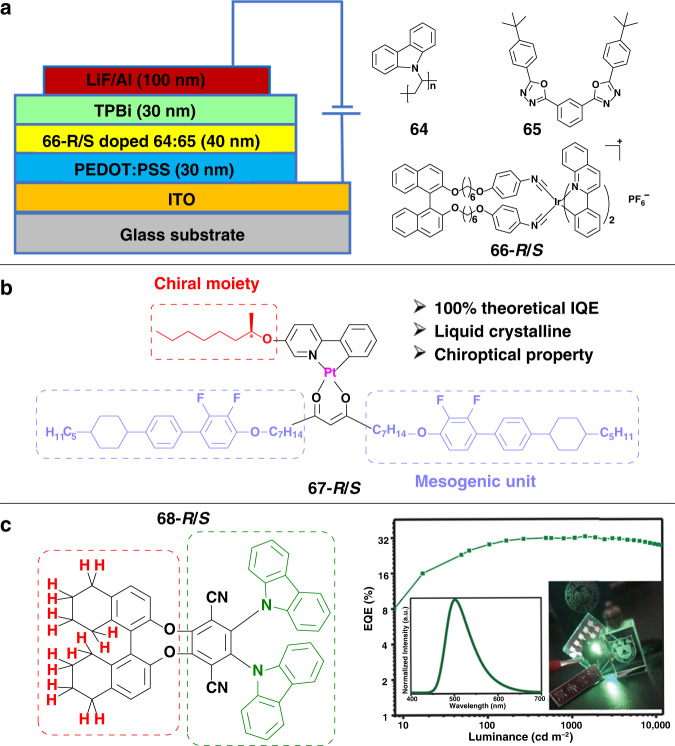
Applications in CP-OLEDs. **a** The device configuration and chemical structures of the chiral Ir(III) complexes. **b** Molecular structure of Pt(II) complexes composed of liquid crystalline moiety and chiral moiety. **c** Chemical structures of the TADF molecules and corresponding EQE-luminance curves of the CP-OLEDs. Images reprinted with permission from: **a** ref. ^117^; **b** ref. ^118^; **c** ref. ^119^ from Wiley

Although some CP-OLEDs with high |*g*_EL_| have been achieved, it is still difficult to obtain CP-OLEDs with both high |*g*_EL_| and external quantum efficiency (EQE). Phosphorescent transition metal complexes have been regarded as promising candidates for CP-OLEDs, which can achieve theoretically 100% internal quantum efficiency by harvesting both singlet and triplet excitons^114–116^. For example, a pair of chiral Ir(III) isocyanide complexes **66** was designed and synthesized by introducing chiral binaphthol into the auxiliary ligand (Fig. 8a)^117^. These two complexes showed quantum yields of up to 21% and 33% in CH_3_CN solution (under argon), and the as-prepared devices exhibited high |*g*_EL_| (the order of 10^−3^). Recently, a pair of cyclometalated Pt(II) complexes **67** was synthesized to fabricate CP-OLEDs (Fig. 8b)^118^. In this system, chiral 2-octanol chain was introduced into the *ortho*-metalated 2-phenylpyridine unit to provide a chiral center, and cyclohexyldifluorobiphenyl derivative was employed as the long-range order of mesogenic unit, which could lead to high carrier mobility. The CP-OLEDs devices exhibited a maximum *g*_EL_ around 0.06 and EQE about 11.3%.

To improve the EQE of CP-OLEDs, another efficient strategy of thermally activated delayed fluorescence (TADF) has also been adopted. TADF materials can convert triplets to singlets through the reversed intersystem crossing, resulting in 100% internal quantum efficiency. The most classical TADF molecules were carbazole-cyan derivatives, and CPL activity can be easily realized by introducing chiral perturbing moieties to the skeleton of TADF molecules, such as binaphthol units. As shown in Fig. 8c, a pair of chiral-emitting enantiomers **68** was designed and synthesized by introducing chiral octahydro-binaphthol^119^. These molecules exhibited high photoluminescence quantum yield of 92%, mirror-symmetric CD signals, and intense CPL activities. Notably, the CP-OLEDs not only achieved intense *g*_EL_ signals of 2.3 × 10^−3^, but also realized high EQE of 32.6% and low efficiency roll-off.

### Optical information processing

**Fig. 9:**
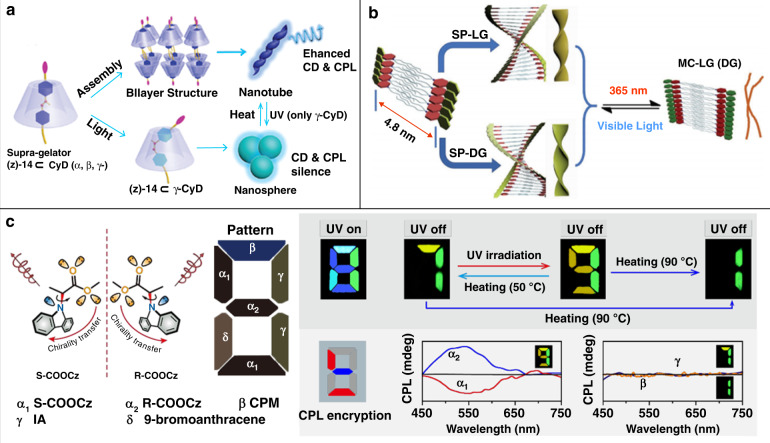
Applications in optical information processing. **a** Schematic illustration of photo-responsive CPL-active supramolecular assembly based on *Z–E* isomerization of cyanostilbene. **b** Schematic illustration of the reversible chiroptical switching of self-assembled spiropyran gels. **c** Illustration of CPL-featured lifetime-encrypted integrated logic device. Images reprinted with permission from: **a** ref. ^120^ from RSC; **b** ref. ^121^; **c** ref. ^125^ from Wiley

Compared with traditional photo-responsive materials, materials with CPL activity can achieve higher storage density and security through optical signals and chiral signals. Therefore, photo-responsive CPL-active materials show broad application prospects in the fields of optical information recording and encryption. Photo-responsive CPL-active materials can be realized by decorating chiral units onto classical photo-responsive materials, such as cyanostilbene, spiropyran, etc. For example, a photo-responsive CPL-active supramolecular assembly was developed based on the *Z–E* isomerization of cyanostilbene (Fig. 9a)^120^. Cyanostilbene was first decorated with chiral glutamate and then incorporated into γ-cyclodextrin via host–guest interaction. After co-assembly with γ-cyclodextrin, the supramolecular gel with nanohelix formed and exhibited enhanced CPL signals. Under UV light irradiation, the *Z–E* isomerization of cyanostilbene occurred, and *E-*state cyanostilbene formed, resulting in nanohelix transformed into nanospheres with silent CD and CPL. By heating, the energetic *E-*state cyanostilbene would return to the stable *Z-*state, and the transformations of both morphological and chiroptical properties could be restored. This reversible *Z–E* isomerization could be repeated at least five cycles by alternating UV light irradiation and heating, exhibiting great potential to develop photo-responsive CPL materials. The ring opening–closure photoisomerization of spiropyran has also been utilized to develop photo-responsive CPL-active materials. Typically, a quadruple optical and chiroptical switch was developed by self-assembly of glutamate gelators containing spiropyran moiety (Fig. 9b)^121^. Under the alternating stimuli of UV and visible light, the spiropyran undergone reversible photoisomerization between ring-closed spiropyran and ring-open zwitterionic merocyanine state. This reversible photoisomerization caused a series of changes in the morphological, optical, and chiroptical properties, realizing reversible quadruple switch (UV–vis absorption, fluorescence, CD, and CPL signals). Furthermore, rewritable printing over 30 cycles without significant loss in contrast and resolution was achieved.

Time-resolved luminescence imaging based on smart organic phosphorescent materials is an emerging and secure optical information encryption and decryption technology^122–124^. Multi-level encryption is expected by integrating CPL and emission lifetime. Recently, a CPL-featured encrypted device was successfully developed based on CP-RTP molecules (Fig. 9c)^125^. By decorating the chiral ester chain onto the carbazole unit, CP-RTP with a lifetime of about 80.0 ms was realized. The flexible ester chain could tune the molecular stacking under UV light irradiation, resulting in photo-activated ultra-long CP-RTP with a lifetime over 0.6 s. The photo-activated ultra-long CP-RTP emissions could also be quickly deactivated to the pristine state by annealing at 50 °C. Benefiting from the reversible photo-activation/thermal-deactivation, a lifetime encrypted logic device was established, and it could be further encrypted by CPL-imaging, realizing multi-level secure encryption. This work provides a new idea for the design of CPL-active materials based secure encryption devices.

### Chemical and biological sensing

**Fig. 10:**
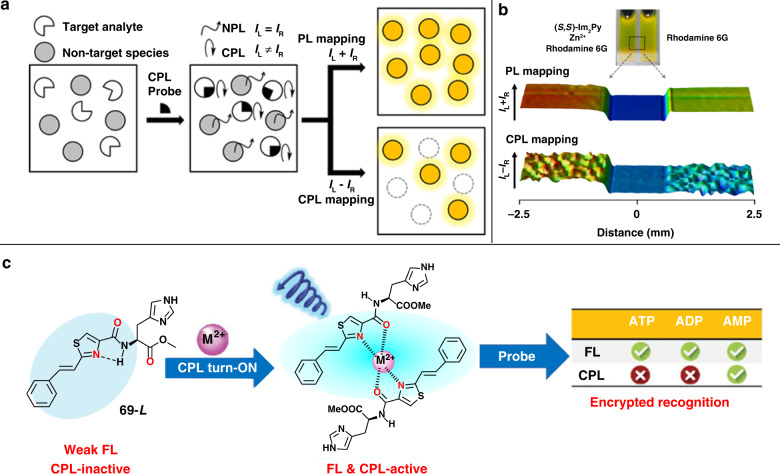
Applications in chemical and biological sensing. **a** Schematic illustration of sensor methodologies based on CPL signal. **b** Photoluminescence mapping and CPL mapping for the target analyte system and non-target specie system. **c** Illustration of encrypted selective recognition of AMP. Images reprinted with permission from: **a**, **b** ref. ^126^; **c** ref. ^131^ from Wiley

Compared with other optical sensing technologies, sensing based on CPL materials can eliminate the interference of background fluorescence and unpolarized light, providing higher sensitivity and resolution. Figure 10a illustrates the mechanism of sensing technology based on CPL signal. In this system, the probe exhibits CPL signal once binding to the target analyte, whereas the background fluorescence and autofluorescence can be eliminated by CPL measurement^126^. Based on this mechanism, a chiroptical probe containing pyrene chromophore and chiral imidazole moieties was developed to detect Zn^2+^. This probe could spontaneously self-assemble into chiral stacks once coordinated with Zn^2+^, and the assemblies showed intense CPL signal. In the object-identification experiment, rhodamine 6G was used as a non-target species because it exhibited similar yellow emission (~ 550 nm) with the assemblies. As shown in Fig. 10b, two samples were prepared: one consists of the probe, Zn^2+^ (target analyte) and rhodamine 6G (non-target specie), while the other contains rhodamine 6G only. Interestingly, there was no obvious difference between these two samples in the photoluminescence mapping. However, the significant difference could be observed in the CPL mapping: intense CPL signal in the system of target analyte, while no CPL signal could be detected in the system of non-target specie only. These results indicate that CPL-based sensing technology provides higher resolution compared with conventional fluorescence sensing. Similarly, highly sensitive sensing of Al^3+^ was developed through the coordination of Schiff bases and metal ions^127^. The chiral Schiff base probe was synthesized by refluxing aromatic aldehydes with amine-terminated glutamine amphiphile, and the probe could self-assemble into nanostructures. Although fluorescence emission and supramolecular chirality were detected, there were no CPL signals for this assembly. In the presence of metal ions such as Mg^2+^, Zn^2+^, and Al^3+^, enhanced fluorescence was observed owing to the inhibition of photo-induced electron transfer and excited-state intramolecular proton transfer. Especially, the CPL signal could be observed in the presence of Al^3+^, realizing highly selective sensing of Al^3+^. In addition, CPL-based sensing technology has also been widely used for selective recognition of bioactive molecules^128–130^. Recently, the selective recognition of adenosine derivatives (ATP, ADP, and AMP) was realized in a chiral self-assembly system composed of histidine derivatives **69** and Mg^2+^ (Fig. 10c)^131^. The assembly system exhibited CPL behavior and could serve as CPL probe. Although the introduction of all the three types of adenosines could enhance the luminescence intensity, ATP and ADP caused the CPL quenching whereas the CPL activity was remained for AMP. This work provides a CPL encrypted detection strategy toward selective recognition of AMP, and will promote the application of CPL probe in sensing of chiral bioactive molecules.

So far, the application of CPL-active organic micro-/nano-structures is still in its infancy. In future research, the development of assemblies with excellent CPL performance is a prerequisite for practical applications. Notably, different applications have different performance requirements. For the application in OLED, it pursues both high quantum efficiency and *g*_lum_, especially in the film sate. In the field of optical information processing, it requires excellent switchable behaviors (rapid responsiveness and repeatability) responsive to external stimuli, whereas good specificity and high *g*_lum_ are expected to provide high sensitivity and resolution for application in chemical and biological sensing.

## Conclusions and perspectives

Organic micro-/nano-structures have been proven to be promising candidates for CPL due to the significantly amplified |*g*_lum_| relative to the single molecule state. In this review, we have briefly summarized the recent progress in CPL-active organic micro-/nano-structures. The performance of assemblies is closely related to both the molecular structures and aggregate structures. Thus, we present the design principle from the aspect of the construction of micro-/nano-structure and the introduction of chirality. In addition, the dynamic nature of assemblies provides an opportunity to regulate the chirality and CPL signal through external stimuli. The high |*g*_lum_| of micro-/nano-assemblies enable the prospects in CP-OLEDs, and the stimuli-responsive properties also make them good candidates for potential applications in optical information processing and sensing.

Although great progress has been made, there are still some challenges. Firstly, the forming process of assemblies is dynamic, which sometimes leads to poor stability or unpredictable performance. Thus, the study of chiral assembly mechanism is needed for designing organic micro-/nano-structures with specific structures and predictable functions. In this process, the dynamic in-situ characterization techniques for the self-assembly process are urgently necessary, especially time-dependent spectroscopy and imaging techniques. Secondly, the current study on the CPL of the micro-/nano-structures is mainly concentrated in solution systems, and the application of CPL-active materials is still in its infancy. There is an urgent need to explore the CPL of the micro-/nano-structures in film state to meet the requirements of optoelectronic applications. The layer-by-layer assembly technique would provide potential approach. The layer-by-layer assembly technique, which is based on intermolecular non-covalent interactions, has been proven to be a simple, versatile and controlled method for the preparation of multilayer thin films. If chiral molecules (or asymmetric environments) are introduced into the assembly process, chirality transfer may occur, inducing the emergence of CPL activity. Thirdly, the self-assembly process often suffers from aggregation-caused quenching, leading to low quantum efficiency. The introduction of AIE effects will provide a general approach for solving the trade-off between large |*g*_lum_| and high quantum efficiency in CPL-active materials.

Finally, an ideal CPL material should simultaneously exhibit large |*g*_lum_| and high quantum efficiency. However, it is difficult to evaluate the comprehensive performance of CPL-active materials due to the trade-off between |*g*_lum_| and quantum efficiency. Recently, the figure of merit (FM) was proposed^92^, which is defined as the product of *g*_lum_ and quantum efficiency. However, the indicator could not intuitively reflect the degree of energy loss. Herein, we propose asymmetric quantum efficiency (*φ*_a_) to evaluate the performance of CPL, and *φ*_a_ is defined as the ratio of left- or right-CPL light intensity (*I*_L_ or *I*_R_) to incident light intensity (*I*_0_).

4 $$\varphi _{\mathrm {a}} = \frac{{I_{\mathrm {L}}}}{{I_0}}\,{\mathrm {or}}\,\varphi _{\mathrm {a}} = \frac{{I_{\mathrm {R}}}}{{I_0}}$$

It can be seen that the larger *φ*_a_ represents lower energy loss, which is of great practical significance. Assuming that the *g*_lum_ is a constant in the whole luminescent spectra and other energy loss is not taken into account, such as reflection and refraction, the *φ*_a_ can be calculated as follows (with the detailed derivation process in support information):

5 $$\varphi _{\mathrm {a}} = \frac{1}{4}\varphi \left( {2 + \left| {g_{{\mathrm {lum}}}} \right|} \right)$$

where *φ* refers to the quantum efficiency.

Overall, it is foreseeable that the study of CPL at micro-/nanoscale will accelerate the development of CPL-active materials. In future work, more efforts should be made to develop CPL-active organic micro-/nano-structures with high *φ*_a_ value and study the mechanism of chirality amplification in self-assembly systems.

## Supplementary information

Supplementary Information
