# Supplementary material for: Circularly polarized luminescence from organic micro-/nano-structures
Source: Light Sci Appl. 2021 Apr 12;10:76. doi: 10.1038/s41377-021-00516-7 (PMC8039044; doi:10.1038/s41377-021-00516-7)
Supplement: Supplementary file 1 — Supplementary Information [file 41377_2021_516_MOESM1_ESM.pdf]

Supplementary Information for

Circularly polarized luminescence from organic micro-/nano-structures

Yongjing Deng,[1] Mengzhu Wang,[1] Yanling Zhuang,[1] Shujuan Liu,[1] Wei Huang,[1,2,*] and Qiang Zhao[1,3,*]

[1] State Key Laboratory of Organic Electronics and Information Displays & Jiangsu Key Laboratory for Biosensors, Institute of Advanced Materials (IAM) & Institute of Flexible Electronics (Future Technology), Nanjing University of Posts & Telecommunications (NUPT), 9 Wenyuan Road, Nanjing 210023, Jiangsu, China.

[2] Frontiers Science Center for Flexible Electronics (FSCFE), MIIT Key Laboratory of Flexible Electronics (KLoFE), Northwestern Polytechnical University (NPU), 127 West Youyi Road, Xi'an 710072, Shaanxi, China.

[3] College of Electronic and Optical Engineering & College of Microelectronics, Jiangsu Province Engineering Research Center for Fabrication and Application of Special Optical Fiber Materials and Devices, Nanjing University of Posts and Telecommunications (NUPT), 9 Wenyuan Road, Nanjing 210023, Jiangsu, China.

*Address all correspondence to Qiang Zhao, E-mail: iamqzhao@njupt.edu.cn; Wei Huang, E-mail: provost@nwpu.edu.cn

Table S1 The approaches to constructing CPL-active organic micro-/nano-assemblies.

Methods		Materials	λ_{em}/nm	φ/%	$\|g_{lum}\|$	φ_a	Application	ref.	Advantages	Disadvantages
Self-assembly of small molecules	Orderly stacking of chiral luminescent molecules	1-*P*	650	24	2.5×10^{-2}	0.122	OLED	38	High $\|g_{lum}\|$; universality	Complicated covalent synthesis; high cost
		2	485	61	2.5×10^{-2}	0.310	CPL switches	47		
		3	500	81.3	3.2×10^{-1}	0.472		48		
		4	487	80.3	5×10^{-2}	0.412		49		
		5-*L*	460		2×10^{-2}			50		
		6	477		3×10^{-2}			51		
		7-*L*	650		1.3×10^{-2}			52		
	Co-assembly of achiral luminescent molecules with chiral molecules	8-*R*, 9	442	42	3.8×10^{-2}	0.214		53	Convenient non-covalent interactions; diversity; versatility; compatibility	Unpredictable performance
		8-*S*, 9	442	43	4.2×10^{-2}	0.220		53		
		10, $EuCl_3$	620		4.4×10^{-3}			54		
		11-*L/D*, 13	460		3×10^{-3}		Chiroptical sensor	55		
		12-*L/D*, 13	470		1.6×10^{-3}			55		
		14-*P*, 15	655		2.67×10^{-4}			56		
		14-*M*, 15	655		1.48×10^{-4}			56		
		16-*L/D*, 17	500		3×10^{-3}			57		
		18-*L*, 19	460		1.3×10^{-3}			60		
		18-*D*, 19	460		1.1×10^{-3}			60		
		20-*R/S*, 21	615	5.2	1.7×10^{-2}	0.026		61		
	Symmetry breaking	22, 23	577		6×10^{-2}			62	Achieving chirality from	Low $\|g_{lum}\|$; poor stability; harsh
		24	427	10.5	8.0×10^{-3}	0.053		42		

									exclusively achiral molecule	preparation conditions
Self-assembly of conjugated polymers	Main-chain chiral conjugated polymers	25-R/*S*	607		1.5×10^{-3}			65	Distinct structure; flexibility uniformity; machinability	Limited axial chiral units; unpredictable performance; high cost
		26-*R/S*	608		None			65		
		27-*R*	~ 500		1.6×10^{-3}			66		
		28-*R*	~ 480		None			66		
		29-*R*	~ 500		None			66		
		30-*R*	~ 500		None			66		
		31-*R/S*	627		1.0×10^{-3}			67		
		32-*R/S*	625		1.8×10^{-3}			67		
		33-*R/S*	622		2.0×10^{-3}			67		
	Conjugated polymers with chiral side-chain	34-*L/D*	600		3.6×10^{-3}		OLED	64	Distinct structure; machinability; tunable properties; uniformity	Complicated synthesis; high cost
		35	500		6×10^{-1}		OLED	68		
		36	498	10.5	2.5×10^{-3}	0.053		69		
		37	498	21.8	7.5×10^{-3}	0.109		69		
		38	600		3×10^{-3}		OLED	70		
		39	615		4×10^{-3}			70		
	Doping achiral conjugated	40, 8-*R*	414		2.4×10^{-2}		OLED	71	High $\|g_{lum}\|$; diversity;	Nonuniformity;
		40, 8-*S*	415		3.1×10^{-2}			71		

	polymers with chiral additives	41-*P/M*, 42	580		5×10^{-1}		OLED	72	flexibility; easy processing	
		42, 43-*R*	546		7.2×10^{-1}		OLED	73		
Self-assembly on micro-/nanoscale architectures	Achiral nanotemplates	44, ZIF-8	475	40	5.5×10^{-3}	0.201	Enantioselective recognition	77	Regular morphologies; good structural stability; controllability	High cost; poor biocompatibility; limited chiral ligands
		(R-MBA)$_2$PbI$_4$	519		1.92×10^{-1}			82		
		(S-MBA)$_2$PbI$_4$	518		2.02×10^{-1}			82		
		(R-MBA)$_4$Cu$_4$I$_4$	630	52.8	1×10^{-2}	0.265		87		
		(S-MBA)$_4$Cu$_4$I$_4$	630	59.7	6×10^{-3}	0.299		87		
		Ag(I) cluster	535		5×10^{-3}			93		
	Chiral nanotemplates	48, DNA	550		1.7×10^{-3}		Biological sensing	97	High preparation efficiency; universality; convenience, diversity; controllability biocompatibility	Poor stability; poor mechanical properties
		49-*L/D*, 50	588	7.5	1.02×10^{-1}	0.039		98		
		51, 52	459	25.1	5×10^{-4}	0.126		99		
		51, 53	495	88.7	2×10^{-4}	0.444		99		
		51, 54	518	33.9	1.3×10^{-3}	0.170		99		
		51, 55	508	28.3	9×10^{-4}	0.142		99		
		51, 56	547	30.0	9×10^{-4}	0.150		99		
		51, 57	590	11.1	1.7×10^{-3}	0.056		99		
		CNCs, 58	502		3.3×10^{-2}		Information	102		

							encryption			
		CNCs/PVA,	480		5×10^{-2}		Information encryption	103		

The detailed derivation process for φ_a

As described in Fig. S1, when chiral luminophore is excited by incident light (I_0), the emitted light can be divided into left- and right-circularly polarized light. If no other energy loss is taken into account, such as reflection and refraction,

$$I_L + I_R = I_0\varphi \tag{S1}$$

According to Equation (1), (4), and (S1), it can be calculated as follow

$$\varphi_a = \frac{1}{4}\varphi(2 + |g_{lum}|) \tag{S2}$$

It can be seen that when the emitted light is unpolarized, $\varphi_a = \varphi/2$ which means the intensity of left and right rotation is equal, and when $g_{lum} = \pm 2$, $\varphi_a = \varphi$ which means completely left- or right-CPL.

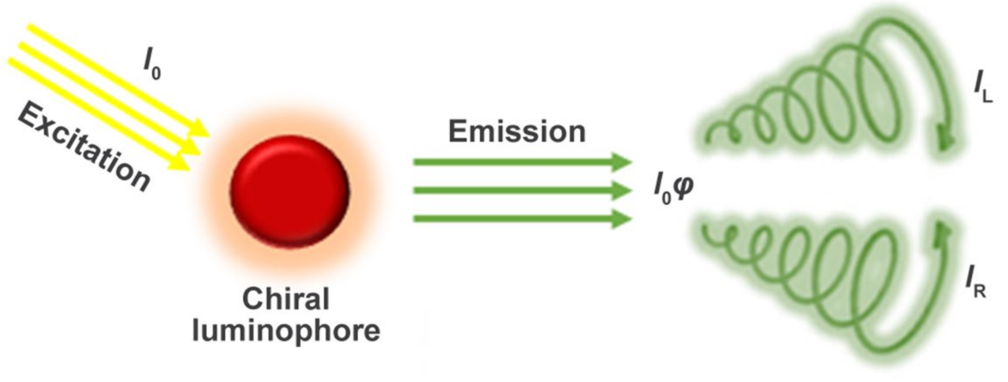

Fig. S1 Relationship of left- and right-CPL intensity, and incident light intensity
